# Long Non-coding RNAs Gabarapl2 and Chrnb2 Positively Regulate Inflammatory Signaling in a Mouse Model of Dry Eye

**DOI:** 10.3389/fmed.2021.808940

**Published:** 2021-12-10

**Authors:** Yuhan Yang, Minjie Chen, Zimeng Zhai, Yiqin Dai, Hao Gu, Xujiao Zhou, Jiaxu Hong

**Affiliations:** ^1^Department of Ophthalmology, Eye and ENT Hospital, Fudan University, Shanghai, China; ^2^Department of Ophthalmology, The Affiliated Hospital of Guizhou Medical University, Guiyang, China

**Keywords:** long non-coding RNA (lncRNA), Gabarapl2 and Chrnb2, dry eye disease (DED), neuroactive ligand–receptor interaction signaling, inflammation

## Abstract

**Purpose:** To elucidate the expression profile and the potential role of long non-coding ribonucleic acids (RNAs) (lncRNAs) in a dry eye disease (DED) model.

**Methods:** A DED model was established in C57BL/6J mice with 0.2% benzalkonium chloride (BAC) twice a day for 14 days. The differentially expressed lncRNAs were detected by RNA-seq technology (Gene Expression Omnibus, GEO GSE186450) and the aberrantly expressed lncRNAs were further verified by RT-qPCR. Gene Ontology (GO) and Kyoto Encyclopedia of Genes and Genomes (KEGG) enrichment analyses were conducted to predicate the related candidate genes and potential pathological pathways. Cells from a human corneal epithelial cell line (HCECs) were cultured under hyperosmolarity. The regulation of inflammatory factors by silencing potential targeted lncRNAs was verified *in vitro* in HCECs.

**Results:** In our study, a significant increase in corneal fluorescence staining and a reduction in tear production were observed in DED mice at all follow-ups compared with the controls, and the differences were increasing over time. In total, 2,649 upregulated and 704 downregulated lncRNAs were identified in DED mice. We selected six aberrantly expressed and most abundant lncRNAs and performed RT-qPCR using the samples for RNA-seq. Chrnb2, Gabarapl2, and Usp31 were thereby confirmed as the most significantly altered lncRNAs. Pathway analysis revealed that the neuroactive ligand–receptor interaction signaling pathway was the most enriched, followed by the calcium signaling pathway and cytokine–cytokine receptor interaction. Following treatment of Gabarapl2 siRNA and Chrnb2 siRNA, tumor necrosis factor-α (TNF-α), interleukin (IL)-1β, and IL-6 were significantly downregulated in the HCECs.

**Conclusion:** Our study suggests that Chrnb2 and Gabarapl2 may be involved in the inflammation response by regulating TNF-α, IL-1β, and IL-6 in DED. These candidate lncRNAs may be both potential biomarkers and therapeutic targets for DED.

## Introduction

Hundreds of millions of people throughout the world are affected by dry eye disease (DED), typically suffering symptoms such as blurred vision, ocular discomfort, and a stinging, burning, scratchy, or gritty sensation while reading, driving, or working with computers (1–5). Unsurprisingly (6), severe symptoms of DED are also associated with decreased work productivity and levels of activity (7). Dry eye is a multifactorial disease of the ocular surface characterized by a loss of homeostasis of the tear film and accompanied by ocular symptoms, in which tear film instability and hyperosmolarity, ocular surface inflammation and damage, and neurosensory abnormalities play etiological roles (5). Numerous cellular and molecular components have been found to contribute to immune-cell activation and associated inflammation in the pathogenesis of dry eye, including inflammatory cytokines, metalloproteinases, and chemokines and their receptors (8–11). Compared to inflammation, neuroinflammation is more persistent in DED environments (12). Nowadays, considerable attention is being paid to the role and mechanisms of ocular-surface nerve inflammation in DED pain sensitivity (13). Neuropathic pain is difficult to cure: all existing therapies, such as serotonin–noradrenaline reuptake inhibitors, anticonvulsants acting at calcium channels, and topical lidocaine and opioids, have only variable success in alleviating pain, while failing to tackle the underlying mechanisms (14, 15).

Long non-coding ribonucleic acids (RNAs) (lncRNAs), which consist of more than 200 nucleotides, are involved in protein translation and messenger RNA (mRNA) decay, which play important roles in regulating inflammatory pathways (16, 17). A growing number of studies has revealed that some lncRNAs participate in the pathogenesis of multiple diseases, including cancer, ocular alkali burns, and Sjögren's syndrome by regulating mRNA directly or indirectly (18–20). Long non-coding ribonucleic acids regulate gene expression through epigenetic regulation, transcriptional regulation, and post-transcriptional regulation, thus participating in various biological processes (BP) such as cell proliferation, differentiation, and apoptosis (21–26). In addition, the interaction of lncRNAs–microRNAs (miRs) participates in the progression of the inflammatory reaction. Myocardial infarction associated transcript 2 (Mirt2) blocks the NF-κB and JAK/STAT signaling pathways by facilitating miR-377 in response to IFN-γ-induced inflammatory insults in Sjögren's syndrome (27). Computational analyses have revealed that differentially expressed lncRNAs are involved in chemokine signaling pathways, the NF-κB signaling pathway, and the tumor necrosis factor (TNF) signaling pathway in labial salivary glands of Sjögren's syndrome patients. However, studies on lncRNAs in the cellular and molecular nerve inflammation pathogenesis of DED are still far from complete.

In the current study, we analyzed the expression profile of lncRNAs in a mouse model of DED using the whole-transcriptome sequencing technology. In our findings, Chrnb2 and Gabarapl2 were confirmed as the most significantly altered lncRNAs. Systemic bioinformatic analyses revealed that the neuroactive ligand–receptor interaction signaling pathway was the most enriched, followed by the calcium signaling pathway and cytokine–cytokine receptor interaction. *In vitro* human corneal epithelial cell line (HCEC) studies revealed that Gabarapl2 siRNA and Chrnb2 siRNA treatment could affect inflammatory factors. Chrnb2 and Gabarapl2 are the potential elements of lncRNAs involved in the pathogenesis of DED, implying a novel target for early therapy.

## Materials and Methods

### DED Mouse Model

Sixty specific-pathogen-free (SPF) C57BL/6J mice (120 eyes), 6–8 weeks old, were randomized into two groups (control group *n* = 25, DED group *n* = 35). The mice were housed in an environmentally controlled room (25°C, with 60% humidity and 12 h/12 h light–dark cycle), with adequate rodent chow and water available. All experimental protocols conformed to the Association for Research in Vision and Ophthalmology statement on the use of animals. We performed surgery on the animals according to the guidelines of the Fudan University Ethics Committee (Ethical code: EENTIRB-2018-03-01). Both eyes in the DED group were administered with 5 μl of 0.2% benzalkonium chloride (BAC) twice a day for 14 days, while phosphate-buffered saline (PBS) was administered to the control group. Schirmer's I-test and the corneal fluorescein sodium staining score were recorded before and at days 7, 14, and 21 after treatment.

### Detection of Basal Tear Secretion (Schirmer's I-Test)

Schirmer's I-test was performed with Zone-Quick Phenol-Red cotton thread. After general anesthesia with 1.25% avertin (0.2 ml/10 g) given intraperitoneally, 1 mm of the folded end of the phenol red cotton line was inserted into the lower lateral conjunctival fornix for 20 s. The color of phenol cotton changes from yellow to red with the secretion of tears. The wetted length (mm) of the strip indicated by red dye was read and recorded. The eyelids were closed after examination.

### Corneal Fluorescein Sodium Staining Score

An experienced oculist took the measurement by a slit lamp with a cobalt blue light. After 90 s of staining with fluorescein sodium, the corneal epithelium was evaluated. We divided the cornea into four quadrants. Each quadrant was checked and the reading recorded. The sum of all quadrants is regarded as the final score. The scoring criteria were: 0 points for no staining; 1 point for fewer than 30 stained dots; 2 points for more than 30 non-diffuse stained dots; 3 points for severe diffuse staining but no plaque staining; and 4 points for a patchy stain.

### RNA-Seq Database Construction Sequencing and Bioinformatics Analysis

Ribonucleic acid was extracted from the corneal tissue of DED mice, and the quality of RNA samples was strictly controlled. Agarose gel electrophoresis was used to analyze the RNA integrity and DNA contamination of the samples. Nanodrop was used to detect RNA concentration and purity. Agilent 2100 BioAnalyzer accurately detects RNA integrity. Ribosomal RNA was removed from total RNA, and then the RNA was broken into short fragments of 250–300 bp. The first complementary DNA (cDNA) strand was synthesized using the fragment RNA as the template and random oligonucleotide as the primer. The RNA strand was degraded by Rnase H. The second strand of cDNA was synthesized from deoxyribonucleotide triphosphates (dNTPs; dUTP, dATP, dGTP, and dCTP) under the DNA polymerase I system. The purified double-stranded cDNA was repaired at the end and “A” tail was added, before sequencing. AMPure XP Beads were used to screen 350–400 bp of cDNA. The second strand of cDNA containing U was degraded by USER enzyme, and PCR amplification was performed to obtain the library. The library was diluted to 1 ng/μl by Qubit. Then Agilent 2100 BioAnalyzer was used to detect the insert size of the library, the insert size being aproximately 250–300 bp. Illumina PE150 sequencing is performed after pooling according to effective concentration and data output requirements with paired-end 150 bp sequencing strategy. After quantitative analysis, the expression matrix of all samples was obtained, and then the significance analysis of expression difference at gene or transcript level was conducted to search for functional genes or transcripts related to the treatment group. EdgeR software was used to analyze the significance of expression differences. The significance level was determined by *p*-value or corrected *p*-value (padj).

### Gene Ontology and Kyoto Encyclopedia of Genes and Genomes Pathway Analyses

In organisms, different genes coordinate their biological functions. Significant pathway enrichment can explore the most important biochemical metabolic pathways and signal transduction pathways involved in differentially expressed genes. ClusterProfiler software was used to select the widely used annotated gene databases Gene Ontology (GO) and Kyoto Encyclopedia of Genes and Genomes (KEGG) for pathway enrichment analysis of differential genes. Gene Ontology enrichment analysis was performed in molecular function (MF), cellular component (CC), and BP. Gene Ontology and KEGG enrichment analysis was performed for co-location and co-expression of differential lncRNAs, respectively, to predict the function of lncRNAs.

### lncRNA–mRNA Co-expression Network

An lncRNA–mRNA expression correlation network was built based on the normalized signal intensity of differential expression in lncRNAs and mRNAs to explore the dysregulation of lncRNAs in DED mice. A co-expression network of control and DED mice was established. Differentially expressed lncRNAs and mRNAs that met the criteria were selected according to *p*-value < 0.05 and fold change >2 or <0.5. Correlations between lncRNA–lncRNA, lncRNA–mRNA, and mRNA–mRNA pairs were assessed using Pearson's correlations.

### Cell Cultures

The HCECs line was kindly provided by Dr. Weiyun Shi (Shandong Eye Hospital, Shandong Eye Institute, Shandong Academy of Medical Sciences, Jinan, China) (28–30). Human corneal epithelial cells were cultured in Dulbecco's Modified Eagle's Medium/Nutrient Mixture F12 Ham (DMEM/F12; Gibco, Carlsbad, CA, USA) containing 100 U/ml penicillin, 100 U/ml streptomycin (Gibco), and 10% heat-inactivated fetal bovine serum (FBS; Hyclone, Rockford, IL, USA). Cells were incubated in a humidified 37°C incubator containing 5% CO_2_.

### Small Interfering RNA Transfection

Chrnb2 and Gabarapl2 knockdown was achieved using small interfering RNA (siRNA). Small interfering RNA were designed and synthesized by RiboBio Technology (Guangzhou, China). Small interfering RNA was transfected using lipofectamine 3000 transfection reagent (Invitrogen, Cat. L3000015; Invitrogen, Waltham, MA, USA) according to the manufacturer's protocol. A total of 5 × 10^5^ cells in 2 ml of medium were seeded in six-well plates. Forty-eight hours post-transfection, cells were harvested in TRIzol for RNA isolation.

### RNA Extraction and RT-PCR Analysis

Total RNA from mice corneal tissue and HCECs were extracted using TRIzol reagent (Invitrogen) according to the manufacturer's instructions, and reverse transcriptions were synthesized using the PrimeScript RT reagent kit (Takara, Japan). RT-PCR analysis was performed using SYBR Premix Ex Taq (Takara) with an ABI Prism 7500 sequence detection system (Applied Biosystems, Waltham, MA, USA). The relative expression of interleukin (IL)-1β, IL-6, TNF-a, Chrnb2, and Gabarapl2 was normalized to the endogenous control GAPDH using the 2^−Δ*ΔCt*^ method.

### Statistical Analysis

Quantitative data were expressed as the mean ± standard error of the mean (SEM). Analysis of variance (ANOVA) was used to test for difference among four different follow-ups, and the Bonferroni test was used to identify which pairs were significantly different. The paired *t*-test or the matched-pairs signed-rank test was used to identify between-group differences. The statistical significance was assumed at *p* < 0.05.

## Results

### DED in the Mouse Model

**Figure 1:**
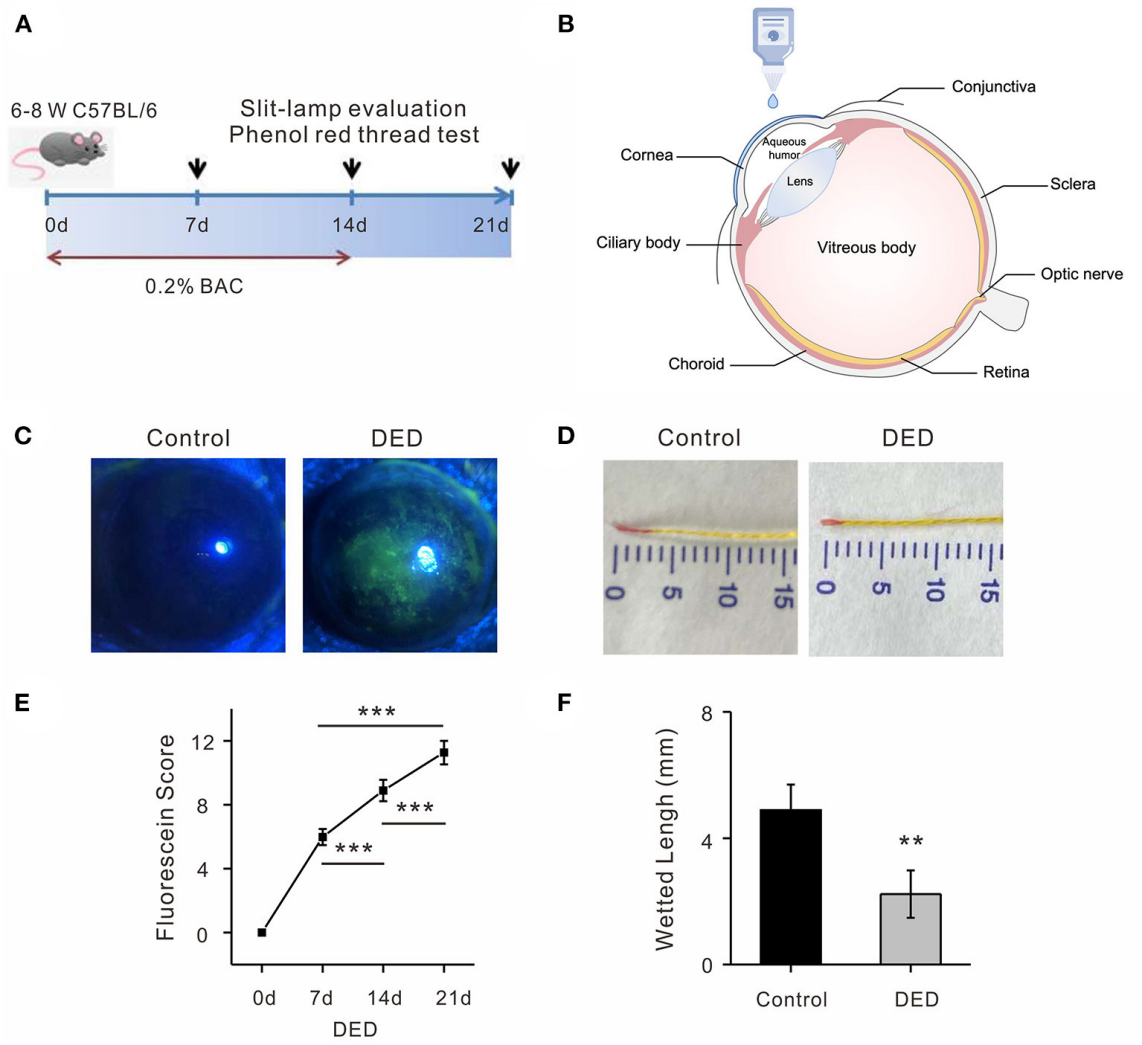
Identification of DED in mouse model. **(A)** Administration of 0.2% BAC in both eyes for 14 days in DED group. Examinations were taken at day 0, day 7, day 14, and day 21. **(B)** Topical 5 μl of 0.2% BAC and PBS were applied twice a day. **(C)** Representative images of fluorescence sodium staining on day 14 in a DED cornea compared with images of the control cornea. **(D)** Representative images of tear production on day 14 after DED animal modeling. **(E)** The fluorescein scores at day 7, day 14, and day 21 were significantly increasing over time in the cornea of the DED mice (all *p* < 0.001). **(F)** Significant reduction of wetted length was observed in the DED group compared with the controls (*p* < 0.01). DED, dry eye disease; BAC, benzalkonium chloride; PBS, phosphate buffered saline.

Seven days after the treatment with 0.2% BAC, obvious epithelial punctate defects were detected using a fluorescence sodium dye in DED mice compared with the control group (Figures 1A–C). We observed no difference in fluorescence score between the two groups by the slit-lamp examination before the treatment (d0) (Figure 1C). The fluorescein scores at day 7, day 14, and day 21 were significantly increasing over time in the cornea of the DED mice compared to control animals (5.98 ± 0.51 at day 7, 8.89 ± 0.67 at day 14, and 11.26 ± 0.74 at day 21; *p* < 0.001; Figure 1E). Tear production was similar before treatment between the two groups of animals (4.89 ± 0.81 mm/30 s). However, there was a rapid and significant reduction in tear production (2.23 ± 0.75, *p* < 0.01, Figures 1D,F) in the DED compared with the control animals.

### Expression Profiling and Bioinformatic Functional Analysis of Differentially Expressed lncRNAs in the Mouse DED Model

**Figure 2:**
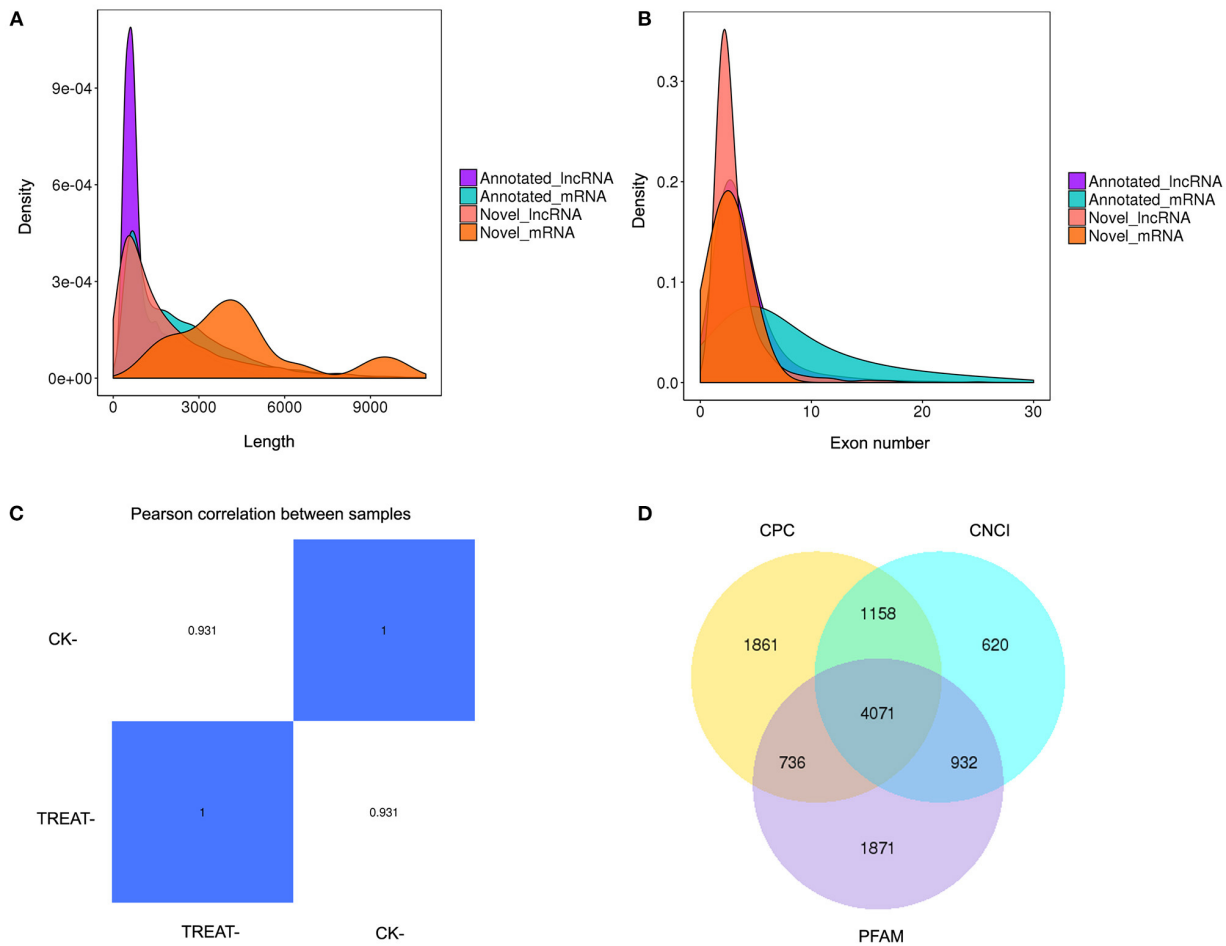
Expression profiling of lncRNAs in the mouse DED model. **(A)** Histogram showing length distribution of novel lncRNA. **(B)** Histogram showing the number of lncRNA exons. **(C)** Heatmap of gene expression correlation; the numerical value refers to the correlation coefficient. **(D)** Venn diagram presenting overlapping potential lncRNAs of three computational approaches (CPC/CNCI/PFAM). DED, dry eye disease; lncRNAs, long non-coding RNAs.

**Figure 3:**
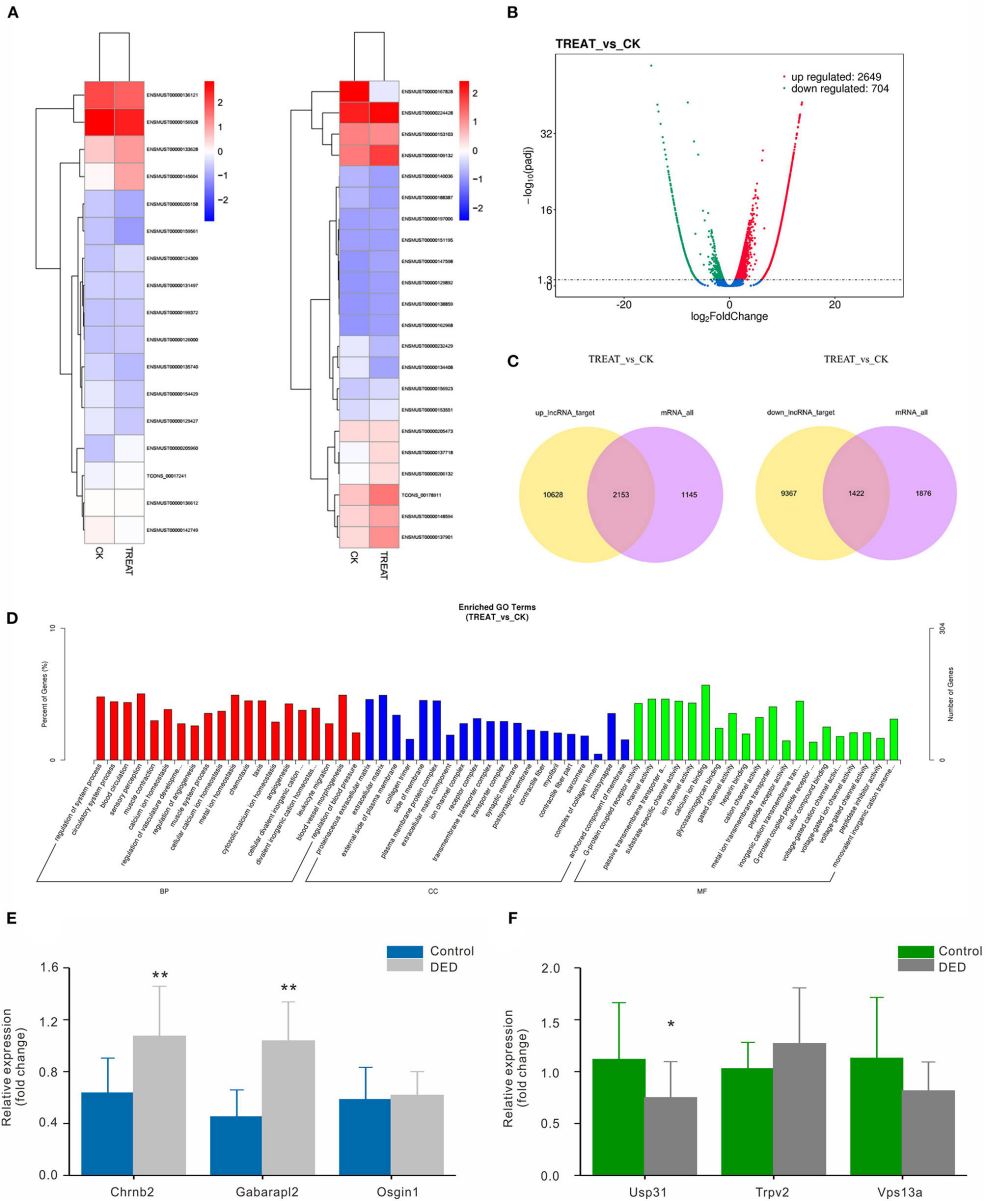
Bioinformatic functional analysis of differentially expressed lncRNAs. **(A)** Heatmap of expression levels of tissue-specific lncRNAs. **(B)** Volcano plot of the different lncRNAs expression in each group. **(C)** Venn diagram showing co-location or co-expression of lncRNA-target genes (mRNAs). **(D)** The number of differentially expressed lncRNAs in BP, CC, and MF. **(E)** Expression levels of the top three upregulated lncRNAs in both groups via RT-qPCR. **(F)** Expression levels of the top three downregulated lncRNAs in both groups via RT-qPCR. CC, cellular component; MF, molecular function; BP, biological process. *p < 0.05, **p < 0.01. DED, dry eye disease.

RNA-seq was performed to identify lncRNAs involved in the cornea with the DED model. The lncRNAs were longer than 200 bp, with more than two exons (Figures 2A,B). The reproducibility between the biological and sequencing technical replicates was very high (Pearson correlation coefficients), at 0.931 (Figure 2C). The Venn diagram indicates the intersection of three computational approaches (CPC/CNCI/PFAM) for candidate lncRNA from putative protein-coding RNAs, and 4,071 aberrantly lncRNAs were predicted as potential lncRNAs using a Venn diagram (Figure 2D). The heatmap of hierarchical clustering revealed that the tissue-specific lncRNA expression patterns among samples were distinct (Figure 3A). Moreover, the Volcano plot revealed the 2,649 upregulated and 704 downregulated lncRNA expression profiles in each group (Figure 3B). Long non-coding ribonucleic acids regulates the expression of target genes (mRNAs) through co-location or co-expression. For the association analysis between lncRNA and mRNA, we performed cross set analysis on target genes with differentially expressed lncRNA and mRNA (Figure 3C). Next, the function of dysregulated lncRNAs and the potential target mRNAs were analyzed. Gene Ontology enrichment analyses were performed. If the Jensen–Shannon (JS) score >0.5, the significantly altered lncRNAs and mRNAs were selected. Subsequently, GO annotation was utilized to investigate the function of differentially expressed lncRNAs, yielding the number in GO terms. In BP, CC, and MF, the numbers of differentially expressed lncRNAs in sensory perception, metal ion homeostasis, extracellular matrix, and calcium ion binding were the highest (Figure 3D). Table 1 shows the top six aberrantly expressed lncRNAs. Table 2 indicates the primer sequences of the top six dysregulated known lncRNAs. In the mice corneal tissue, we evaluated the differences between groups with these six select genes by RT-qPCR (Figures 3E,F). The results showed that cholinergic receptor nicotinic beta 2 (Chrnb2) and autophagy target factor (gamma-aminobutyric acid (GABA) a receptor-associated protein-like 2, Gabarapl2) were significantly increased following DED compared with the control group (*p* < 0.01) (Figure 3E). Then, we verified the between-group differences with the top three downregulated lncRNAs, revealing significantly decreased expression levels of Usp31 (*p* < 0.05) (Figure 3F).

**Table 1:** The list of the top 6 dysregulated known lncRNAs.

**Transcript ID**	**Gene name**	**Regulation**	**Log2 fold change**	**Locus**	**TREAT**	**CK**	***p*-Value**
ENSMUST00000199372	Chrnb2	Up	6.950176971	3:89752433–89763401	0.457683	0.002203	4.23E-21
ENSMUST00000133628	Gabarapl2	Up	2.105375715	8:111941068–111952742	3.161783	0.674989	1.15E-06
ENSMUST00000145604	Osgin1	Up	2.904841522	8:119437167–119446256	2.935011	0.360286	1.35E-10
ENSMUST00000205473	Usp31	Down	−1.895615293	7:121658782–121666815	0.403898	1.383633	1.72E-05
ENSMUST00000151195	Trpv2	Down	−10.13605601	11:62574486–62600299	0	0.093729	1.27E-17
ENSMUST00000224428	Vps13a	Down	−2.173306821	19:16734025–16780912	1.426892	5.919149	3.96E-07

**Table 2:** Primers used in RT-PCR.

**Transcript ID**	**Forward primer**	**Reverse primer**
ENSMUST00000199372	TGCTGACGGCATGTACGAAG	TGCTGGTCAAATGGGAAGTG
ENSMUST00000133628	TCACTGTGGCTCAGTTCATG	TAGTTAGGCTGGACTGTGGG
ENSMUST00000145604	CTCACATTAGACCCGTGCTTC	GACTGTCACTGTGGTCCCTCT
ENSMUST00000205473	GGAAGGAGACAGGCGTATGA	GGGACCAATGAGATGGCAAG
ENSMUST00000151195	TGAGGCTTAGACAGCGTGAG	GGTAGTTCTTCATCCCAGAGG
ENSMUST00000224428	GAGTGGTGGGCTTATGCTAT	TTCTTTGCCTCAACTTCTGC
GAPDH	GCACCACACCTTCTACAATG	GTGAGGGAGAGCATAGCC

### KEGG Pathway Analysis and Regulatory Mechanism of Differentially Expressed lncRNAs

**Figure 4:**
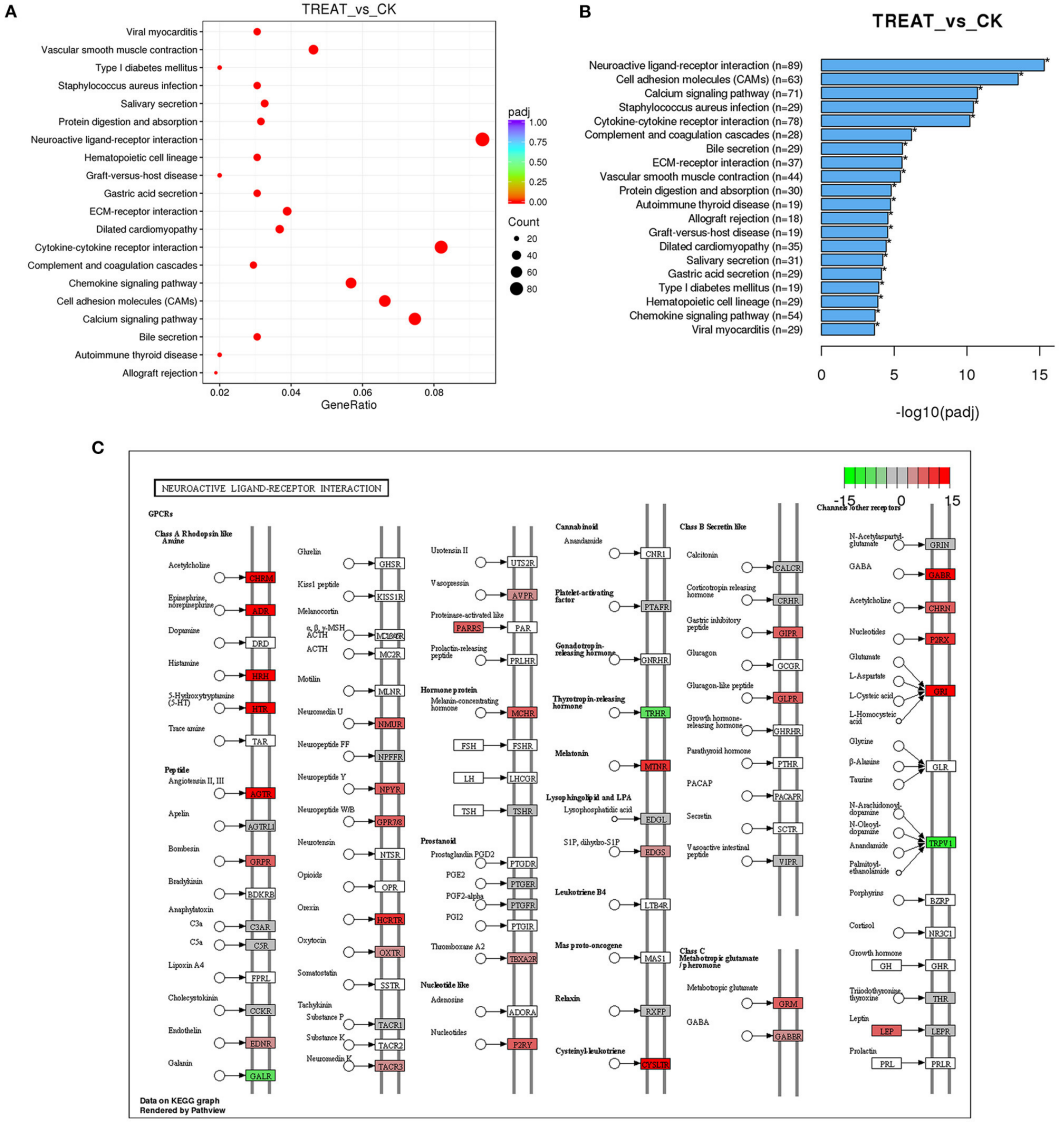
Signaling pathway analysis of differentially expressed lncRNAs. **(A)** The KEGG pathway analysis demonstrates that the genes are enriched in multiple pathways. **(B)** Lists of the most affected pathways. **(C)** Neuroactive ligand–receptor interaction signaling pathway map. Red in **(C)** indicates upregulated genes; green in **(C)** indicates downregulated genes; lncRNAs, long non-coding RNAs; KEGG, Kyoto Encyclopedia of Genes and Genomes.

Multiple active pathways were found in expressed lncRNAs by KEGG pathway analysis. Among them, the neuroactive ligand–receptor interaction signaling pathway was the most enriched, followed by cytokine–cytokine receptor interaction, the calcium signaling pathway, and the cell adhesion molecules (CAMs) signaling pathway (Figures 4A–C). Based on the above results, Chrnb2 and Gabarapl2 were the most significantly altered lncRNAs and may play a critical role in the molecular network of DED.

**Figure 5:**
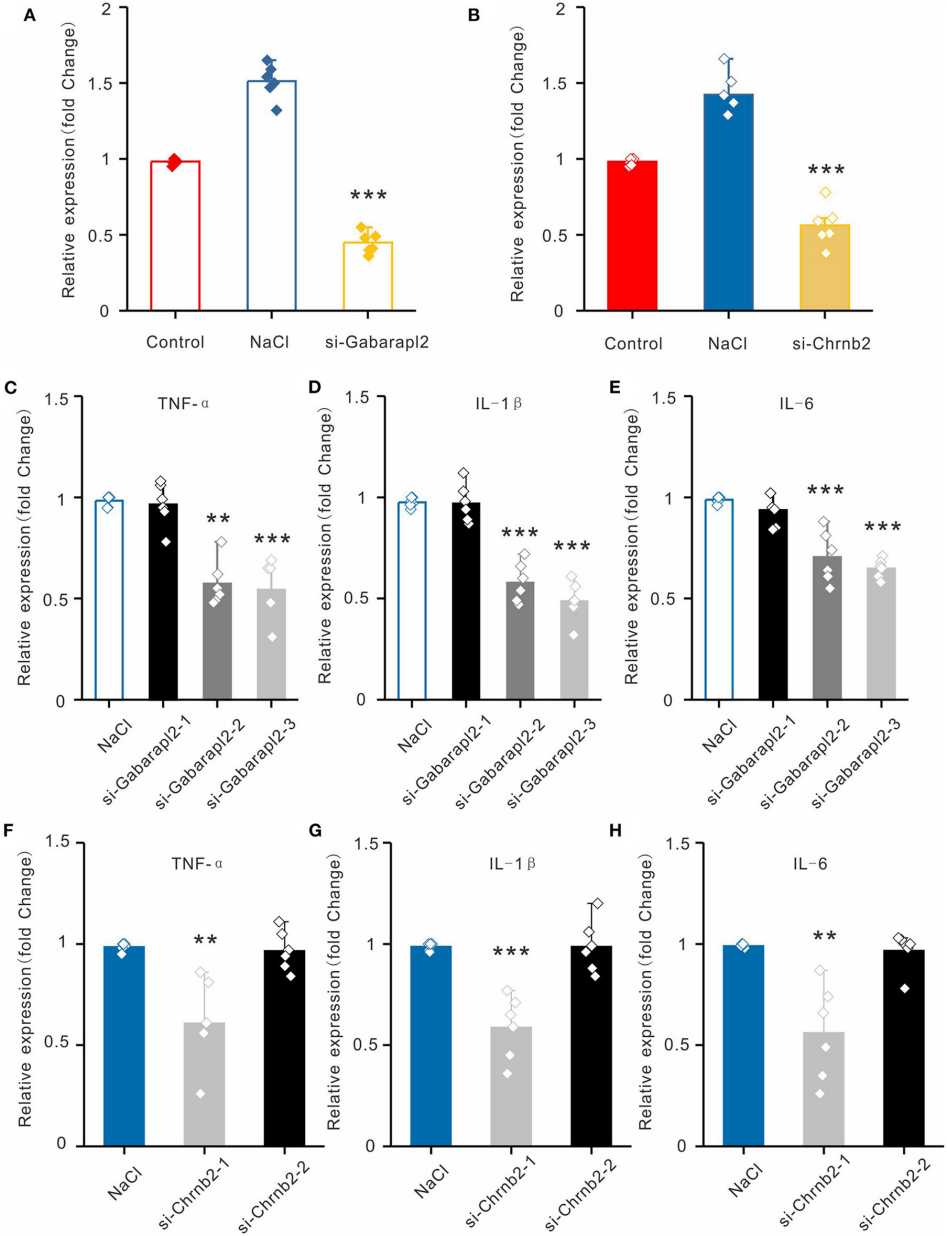
Validation of lncRNAs Chrnb2 and Gabarapl2, regulating the inflammatory factor *in vitro*. **(A,B)** The expression levels of Chrnb2 and Gabarapl2 in HCECs after NaCl treatment. **(C–E)** After transfection with si-Gabarapl2, the mRNA levels of TNF-α, IL-1β, and IL-6 in HCECs following NaCl treatment. **(F–H)** After transfection with si-Chrnb2, the mRNA levels of TNF-α, IL-1β, and IL-6 in HCECs following NaCl treatment. **p < 0.01, ***p < 0.001.

To establish the hyperosmolarity-induced HCECs dysfunction model, we exposed HCECs to sodium chloride (NaCl) at a concentration of 100 mM for 6 h. For further corroboration, we compared the expression levels of the two candidate lncRNAs in HCECs with or without NaCl treatment (the inflammation DED model *in vitro*). Consistent with the qRT-PCR results in the mice cornea *in vivo*, Chrnb2 and Gabarapl2 were remarkably elevated after NaCl treatment in HCECs (Figures 5A,B). To verify that candidate lncRNAs are involved in inflammatory insults, siRNA-mediated knockdown experiments on the two lncRNAs were performed *in vitro* in HCECs by transfection. The two candidate lncRNAs were designed to produce three siRNAs (Gabarapl2: si-Gabarapl2-1, si-Gabarapl2-2, si-Gabarapl2-3) and two siRNAs (Chrnb2: si-Chrnb2-1, si-Chrnb2-2) according to primer sequence, respectively. The culture supernatant was collected for monitoring the inflammatory cytokines. As shown in Figures 5C–H, the expression levels of TNF-α, IL-1β, and IL-6 were significantly reduced in HCECs after transfection with si-Gabarapl2-2/si-Gabarapl2-3 (si-Gabarapl2-2: 0.98 ± 0.03 vs. 0.57 ± 0.11 in TNF-a, *n* = 6, *p* < 0.001; 0.98 ± 0.02 vs. 0.58 ± 0.10 in IL-1β, *n* = 6, *p* < 0.001; 0.99 ± 0.02 vs. 0.71 ± 0.13 in IL-6, *n* = 6, *p* < 0.001. si-Gabarapl2-3: 0.98 ± 0.03 vs. 0.54 ± 0.15 in TNF-a, *n* = 6, *p* < 0.001; 0.98 ± 0.02 vs. 0.49 ± 0.10 in IL-1β, *n* = 6, *p* < 0.001; 0.99 ± 0.02 vs. 0.65 ± 0.05 in IL-6, *n* = 6, *p* < 0.001), or si-Chrnb2-1 (si-Chrnb2-1: 0.99 ± 0.02 vs. 0.61 ± 0.21 in TNF-a, *n* = 6, *p* < 0.01; 0.98 ± 0.02 vs. 0.59 ± 0.16 in IL-1β, *n* = 6, *p* < 0.001; 0.99 ± 0.01 vs. 0.56 ± 0.24 in IL-6, *n* = 6, *p* < 0.01) in HCECs. The above results suggested that Gabarapl2 and Chrnb2 silence significantly repressed TNF-α, IL-1β, and IL-6.

## Discussion

Dysfunction of lncRNAs is highly associated with various human physiological processes, such as cell proliferation, development, metastasis, apoptosis, and immune response modulation (31–33). Additionally, the critical role of lncRNAs in the pathogenesis and regulation of ocular disorders such as glaucoma and cataract has been well-established (34, 35). In glaucoma, lncRNA-MALAT1 could inhibit retinal ganglion cell (RGC) apoptosis through activation of the PI3K/Akt signaling pathway (35). MIAT was found to act as a competing endogenous RNAs (ceRNA) and to form a feedback loop with Akt and miR-150-5p to inhibit TNF-α-induced proliferation and migration of human lens epithelial cells (HLECs) under oxidative stress (34). Subsequently, we explored the potential targeted lncRNA expression profile in a mouse model of DED. In the present study, we identified 3,353 significantly dysregulated lncRNAs in DED, further confirmed by PCR analysis. In total, 2,649 upregulated and 704 downregulated co-expressed lncRNAs were identified in the DED model. Notably, Chrnb2, Gabarapl2, and Usp31 were verified as the potential regulated lncRNAs compared with the control group. Knockdown of Chrnb2 or Gabarapl2 alleviated the pro-inflammatory signals in HCECs in the hypertonic state. All these results indicated that lncRNAs may participate in the mechanisms of DED.

Our correlation analysis between differentially expressed lncRNA and target gene mRNA showed that the Chrnb2 and the autophagy-related gene (GABA, a receptor-associated protein-like 2, Gabarapl2) were significantly up-regulated. Few studys have yet linked them to the eye diseases. Previous studies had found that Chrnb2 gene mutations induce autosomal dominant nocturnal frontal lobe epilepsy (36). Mice lacking expression of Chrnb2 display abnormal retinal waves and a dispersed projection of RGC axons to their dorsal lateral geniculate nuclei (dLGNs) (37). Gabarapl2 belongs to autophagy-related 8 (Atg8) gene family member. Autophagy-related 8 regulates GABA receptor-associated protein, GABARAP, GABARAP is one of the earliest and most important helper protein that mediates GABA_A_ receptors transport from Golgi apparatus to the cell membrane (38, 39). In mammals, Atg8 has evolved into the LC3/Gabarap protein family, which consists of LC3A, LC3B, LC3C, Gabarap, Gabarapl1, and Gabarapl2 (40, 41). Sasau et al. revealed that Gabarap is involved in host immunization system, especially interferon-inducible GTPases (41).

Furthermore, we performed GO and KEGG enrichment analyses to explore signaling pathways and their relationship with biological systems, revealing that the neuroactive ligand–receptor interaction signaling pathway was the most significant pathway. This pathway was previously evidenced as functionally significant in neurotransmitter-mediated disorders such as alcohol dependence disorder (42), autism spectrum disorders (43), Parkinson's disease (44), and some types of lung cancer (45). Besides, the pathway was found to be enriched in proliferative diabetic retinopathy and age-related macular degeneration (46, 47). This pathway regulates multiple neuroreceptors and their associated distant signaling molecules, such as 5-hydroxytryptamine (5-HT, serotonin) and GABA. A previous study found DED patients have higher tear serotonin levels (48), whereas another study indicated that selective serotonin reuptake inhibitor (SSRI) promotes an inflammatory response on the ocular surface by increasing the tear serotonin levels (49). It may be concluded that the serotonin acted as a mediator during the etiology of DED. However, the upstream and downstream mechanisms of serotonin in DED have not been fully elucidated. Another parallel study on the mechanism of serotonin regulation of dry eye is ongoing. The enriched expression of GABA in the neuroactive ligand–receptor interaction signaling pathway hints toward further understanding. Researchers demonstrated that GABA- and 5-HT-immunoreactive neurons could constitute parallel inhibitory or excitatory pathways (50, 51). Further, GABA can regulate the release of serotonin and modulate serotonergic neurons in the nervous system (52, 53). Meanwhile, as the neurotransmitters, 5-HT and GABA usually participate in the physiological cycle together (54). According to the definition of DED by the Tear Film and Ocular Surface Society (TFOS) Dry Eye Workshop (DEW) II, the core mechanism of DED is tear hyperosmolarity, which is the hallmark of the disease (5). Tear hyperosmolarity, as well as inflammatory mediators, may induce DED symptoms and cause damage to the ocular surface, including epithelial cells, surface microvilli, and goblet cells (5, 11). Some lncRNAs can interact with RNA binding proteins (RBPs) and promote the release of corresponding cytokines (55–57), including IL-6, TNF-α, and IL-1β, which may play a significant role in the regulation of DED (58). Long-term and recurring nerve inflammations can also create a chain effect. The neuroactive ligand–receptor interaction signaling pathway may regulate the inflammatory process. 5-Hydroxytryptamine-related GABA regulatory enhances the phagocytic functions of monocytes/macrophages and induces them to produce pro-inflammatory cytokines, stimulating neutrophil chemotaxis (59). Thus, we speculated that tear hyperosmolarity could trigger 5-HT and GABA secretion, which stimulates production of pro-inflammatory cytokines, resulting in increased friction and also symptoms.

Meanwhile, the results showed that the cytokine–cytokine receptor interaction signaling and calcium signaling pathway were also enriched in the DED model. Nociceptors respond to cytokines and produce inflammatory mediators. Non-neuronal cells release pro-inflammatory mediators such as TNF and IL-1β, which live in close proximity to nociceptors following injury or insult, promoting pain transduction via the modulation of ion channels (transient receptor potential vanilloid 1, TRPV1) (12, 60–62). The expression levels of a lncRNA (uc.48+) were increased in the dorsal root ganglions (DRGs) of diabetic rats. Knockdown of uc.48+ alleviated TNF-α in DRGs of diabetic rats (63). A significant upregulation of genes associated with inflammatory and immune responses was found to be related to cytokine–cytokine receptor interactions in human corneal, conjunctival, and meibomian glands (64). Moreover, in a DED-related therapeutic study, diquafosol promoted corneal epithelial healing via intracellular calcium-mediated signaling (65). These findings indicate that both cytokine–cytokine receptor interaction signaling and calcium signaling pathways may participate in the inflammatory process of DED. Consistent with previous studies, our results also showed that the expression levels of Chrnb2 and Gabarapl2 were upregulated in HCECs after NaCl treatment, and mice corneas of DED and knockdown of Chrnb2 and Gabarapl2 could alleviate the expression of TNF-α, IL-1β, and IL-6 *in vitro* and *in vivo*. However, the exact molecular mechanism contributing to the etiology of DED is not fully defined, warranting further exploration.

In conclusion, our study first reported the lncRNA high-transcriptome sequencing analysis of DED. In view of our results, 3,353 dysregulated lncRNAs were explored. Among them, Chrnb2, Gabarapl2, and Usp31 were verified as potentially regulated lncRNAs. Further, we speculated that the neuroactive ligand–receptor interaction signaling pathway may be involved in the inflammatory process of DED via neurotransmitter secretion. TNF-α, IL-1β, and IL-6 were significantly reduced in HCECs after transfection with si-Chrnb2 or si-Gabarapl2. Thus, the current findings may lead to a greater depth of understanding of DED, providing potential regulatory mechanisms of lncRNAs in the DED and therapeutic targets based on the differentially expressed lncRNAs.

## Data Availability Statement

The datasets presented in this study can be found in online repositories. The names of the repository/repositories and accession number(s) can be found below: https://www.ncbi.nlm.nih.gov/geo/, GSE186450.

## Ethics Statement

All experimental protocols conformed to the Association for Research in Vision and Ophthalmology statement on the use of animals. We performed surgery on the animals according to the guidelines of the Fudan University Ethics Committee (Ethical code: EENTIRB-2018-03-01).

## Author Contributions

YY and MC performed the *in vitro* experiments and analyzed the data. ZZ and YD performed the *in vivo* experiments. XZ and JH designed and supervised the project, and wrote the manuscript. All authors contributed to manuscript revision, read, and approved the submitted version.

## Funding

This work was financially supported by the National Natural Science Foundation of China (81970766 and 82171102), the Program for Professor of Special Appointment (Eastern Scholar) at Shanghai Institutions of Higher Learning, the Shanghai Innovation Development Program (2020-RGZN-02033), the Shanghai Key Clinical Research Program (SHDC2020CR3052B), and the Natural Science Foundation of Shanghai (19ZR1408400).

## Conflict of Interest

The authors declare that the research was conducted in the absence of any commercial or financial relationships that could be construed as a potential conflict of interest.

## Publisher's Note

All claims expressed in this article are solely those of the authors and do not necessarily represent those of their affiliated organizations, or those of the publisher, the editors and the reviewers. Any product that may be evaluated in this article, or claim that may be made by its manufacturer, is not guaranteed or endorsed by the publisher.
