# Supplementary material for: Long Non-coding RNAs Gabarapl2 and Chrnb2 Positively Regulate Inflammatory Signaling in a Mouse Model of Dry Eye
Source: Front Med (Lausanne). 2021 Dec 10;8:808940. doi: 10.3389/fmed.2021.808940 (PMC8703135; doi:10.3389/fmed.2021.808940)
Supplement: Supplementary file 8 [file Data_Sheet_1.PDF]

Supplementary Material

Long non-coding RNAs Gabarapl2 and Chrnb2 positively regulate inflammatory signaling in a mouse model of dry eye

Yuhan Yang[1,2]†, Minjie Chen[1]†, Zimeng Zhai[1], Yiqin Dai[1], Hao Gu[2], Xujiao Zhou[1]*, Jiaxu Hong[1,2]*

**Correspondence:* Xujiao Zhou, Jiaxu Hong

xujiaozhou@126.com

jiaxu.hong@fdeent.org

Supplementary Figure

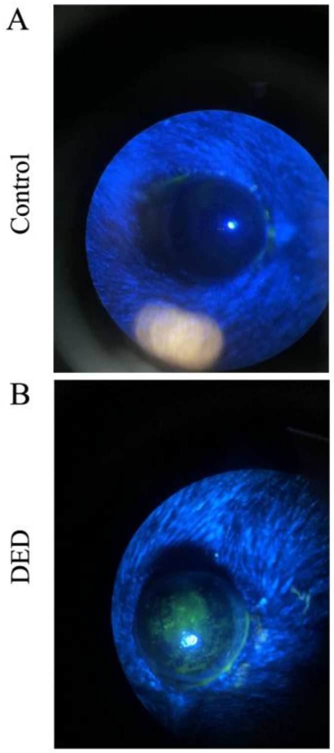

Supplementary Figure S1. Original image taken under a Zeiss microscope after staining with sodium fluorescein. **(A)** Control corneal imaging. **(B)** DED corneal imaging.
