# Supplementary figures and images for: Long Non-coding RNAs Gabarapl2 and Chrnb2 Positively Regulate Inflammatory Signaling in a Mouse Model of Dry Eye
Source: Front Med (Lausanne). 2021 Dec 10;8:808940. doi: 10.3389/fmed.2021.808940 (PMC8703135; doi:10.3389/fmed.2021.808940)

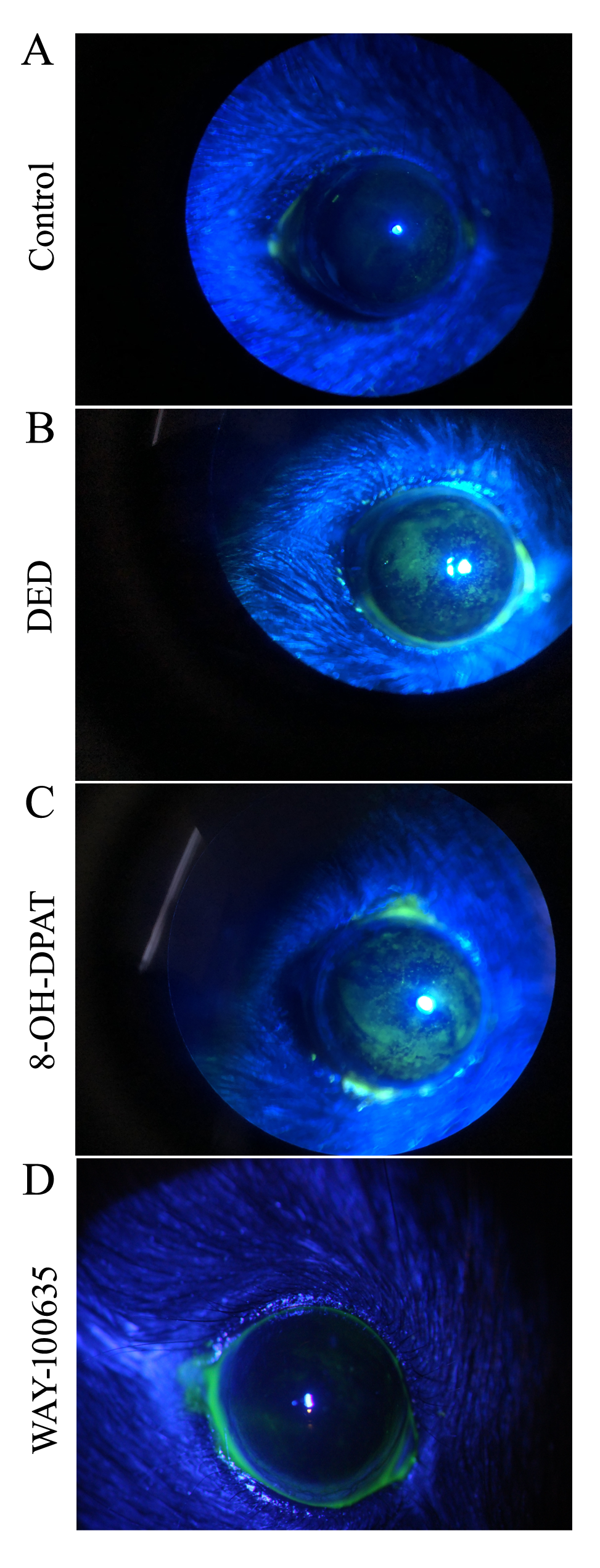

Supplement: Supplementary file 9 [file Image_1.TIF]
